# Visual Signaling in the Semi-Fossorial Lizard *Pholidobolus* *montium* (Gymnophthalmidae)

**DOI:** 10.3390/ani11113022

**Published:** 2021-10-21

**Authors:** Franco Poma-Soto, Andrea E. Narváez, Andrés Romero-Carvajal

**Affiliations:** 1 Escuela de Ciencias Biológicas, Pontificia Universidad Católica del Ecuador, Quito 170525, Ecuador; 2 Fundacion Great Leaf, De las Azucenas N47-60 y Av. Eloy Alfaro, Quito 170503, Ecuador

**Keywords:** *Pholidobolus*, lizard, behavior, visual signaling

## Abstract

**Simple Summary:**

Lizards display multiple communication modalities, through chemical, visual, vocal, or tactile signals which mediate sociality, reproduction, territoriality, competition, and other complex interactions among individuals. In some species that dwell on the surface, it has been shown that multimodal communication is possible, for example, visual and chemical communication. It is less known if lizards that dwell in caves or burrows (fossorial) also use visual signals. By studying behavior in a semi-fossorial lizard from the northern Ecuadorian Andes, we have discovered that they can use visual signals like leg movements and body arching to communicate. In this manuscript, we describe these observations and discuss the potential roles of these signals. This is the first description of such behaviors in semi-fossorial lizards.

**Abstract:**

It has been suggested that gymnophthalmids, like most semi-fossorial lacertoids, rely more in chemical cues to communicate, in comparison to other groups, like Iguanids, on which communication is mostly based on visual signaling. We present the first description of visual signaling in the Andean lizard *Pholidobolus* *montium* (Gymnophthalmidae) and a complete ethogram based on ex situ observations (34 different types of behaviors including positions and simple movements). Through the design of conspecific stimulus experiments, we were able to recognize leg-waving as a visual signal, as it is only displayed in presence of conspecifics or in presence of a mirror and was one of first and most frequent displays in this context. We also detected other visual displays like neck-arching and tail-undulation which may also be relevant as visual signals. Based on our results, we propose that visual signaling is also possible in semi-fossorial lizards; however, further studies regarding chemical signal recognition and color detection are required to confirm our hypothesis.

## 1. Introduction

Communication signals vary greatly across squamate reptiles and may involve acoustic, visual, and chemical components [1,2,3,4,5,6,7]. Chemical communication in lizards comes by means of the secretions of chemical signals and its vomerolfactory reception [5]. Chemical signals are secreted by epidermal glands such as the femoral follicular glands and the preanal glands, although metabolites in skin or in feces may also be important [8,9,10]. In addition to chemical signaling, visual signaling is widespread across lizards and consists of the context-dependent presentation of visually distinctive skin ornaments like dewlaps, proboscides, colorful parts of the body, changes in coloration, movement of the head, limbs or tail, among others [11].

Historically, it has been suggested that some groups within Squamata, like Gymnophthalmidae, rely more in chemical signaling as a form of communication, compared to other clades on which communication is mostly based on visual signaling [12,13,14,15]. However, epidermal glands and chemoreception ability (judged by vomerolfaction development, tongue shape, and tongue-flicking) are present in every lizard clade; therefore, it is possible that most lizards use multiple signal modalities (chemical and visual signaling, for example) to communicate [9,14,16,17,18]. It is less clear if fossorial or semi-fossorial lizards also use visual signaling. Indeed, these species usually lack dewlaps or other visible accessorial structures commonly associated with visual communication [19].

Gymnophthalmids are lizards with cryptic dorsal color patterns, and fossorial or semi-fossorial behaviors [20]. These characteristics, in addition to a “non-charismatic” status, have limited studies regarding its biology and natural history. In gymnophthalmid lizards, as well as in other fossorial lacertoids, it has been suggested that communication is conducted mostly using chemical cues to inform behaviors such as social interactions, courtship, and mating [14,18]. According to the most complete evolutionary analysis of lizard display behaviors, produced by Johnson et al. [11], within the family Gymnophthalmidae there are no records of visual displays that involve postural changes, movement of head, limbs or mandible, and very few species (only 2 out of 31 genera analyzed) show stimulus elicited tail movements. Importantly, a few species of Gymnophthalmidae have conspicuous colorations while some others exhibit color bands or color patches in the belly, throat, or limbs [21], yet the signaling value of such characteristics also remains to be studied. 

*Pholidobolus montium* [22] is a small (SVL: male 56 mm, females 66 mm) diurnal terrestrial gymnophthalmid lizard with grey-black dorsal iridescent color patterns, pale yellow dorsolateral stripe, and a white creamish lip stripe extended towards the forelimb. This species is present in the highlands of the northern Ecuadorian and southern Colombian Andes [23], and it is commonly named “cuilán” or “miner lizard” (Lagartija minadora) as it hides in natural or man-made burrows, open shrubby areas, rock piles, stone walls, and agave fence rows [24,25]. *Pholidobolus montium* has been described previously as a diurnal species with foraging and basking habits [25]. Burrows seem to be the most preferred site for nesting for *P. montium*, where females deposit two-egg clutches per reproductive cycle, with previous reports suggesting there is continuous breeding throughout the year [25,26,27]. Furthermore, Ramirez-Jaramillo [26] reported two communal nests for this species, which may be evidence of social aggregation and other complex social interactions [28]. Remarkably, *P. montium* and *Pholidobolus prefrontalis*, two of the fourteen species of *Pholidobolus*, lack femoral follicular glands [21,23,25]. However, nothing is known regarding communication and signaling in this species. *Pholidobolus montium* is also a “near threatened” species that is disappearing from populated areas where it used to thrive in previous decades [29]; thus, ex situ studies of *P. montium* reproduction and behavior are sorely needed.

To assess the suitability of ex situ breeding and maintenance of *P. montium*, we generated a complete description of *P. montium* behavior in captivity through daily observation and the recording of behavioral patterns of individuals. This description includes an ethogram generated from the constant observation of isolated adult males and females, and the behaviors observed in a focal lizard in the presence of conspecifics. In this context, we were able to record, for the first time, conspecific elicited visual displays in a gymnophthalmid lizard. Finally, we discuss the functional significance of this display relative to the context in which it occurs.

## 2. Materials and Methods

### 2.1. Animal Collection and Housing

Animal handling protocols and procedures were designed following the Guidelines for Use of Live Amphibians and Reptiles in Field Research [30]. *Pholidobolus montium* adults were captured at the urban locality of Calacalí (0°00′00.4″ S, 78°30′39.4″ W, 2819 m; T = △12.5 °C) in the northern Ecuadorian highlands. We located the lizards by active search, collected them by hand, and placed them in separate plastic containers within a cooler. The collection points are urbanized areas designed for outdoor sports with constant human presence. Sixteen individuals (7 males and 9 females) were captured in August 2016 and nineteen individuals (9 males and 10 females) during September 2017. Sex was determined by two criteria: males are more swollen at the base of the tail than females, and a brownish spot at both sides of the head is observable in some males but never on females. Only adults were collected, based on sexual dimorphic characters and on the adult snout-vent length or SVL (males = 44.31 + 2.14 mm, females = 48.1+ 1.92 mm), which limited drastically the number of collected individuals. All individuals were transported in plastic tubs to the laboratory at Pontificia Universidad Católica del Ecuador (PUCE) in Quito, 23 km from the collection site. Each lizard was assigned a code which consisted of a number and information related to its collection site and sex. Lizards were maintained in the laboratory until August 2018, when they were released to the same collection site. 

Lizards were housed in a semi-closed greenhouse where temperature was not controlled and fluctuated with the weather outside. The average temperature recorded in the green house was 19 °C + 6.33 °C. Lizards were isolated individually in glass terrariums without lid (50 cm × 25 cm × 30 cm). Illumination was provided by natural light from windows and white-light fluorescent bulbs (60 Watts) suspended 20 cm above the terrarium and controlled with a timer to obtain a 12 h. light–dark cycle (06:30–18:30). Each terrarium contained a substrate of gravel (12 mm diameter), permanent supplement of water in a plastic petri dish, and an inverted dark plastic plate (13 cm diameter) used as burrow. Terrariums were misted daily, and lizards were fed appropriately sized crickets dusted with multivitamin powder three times weekly. 

### 2.2. Behavioral Observations

To observe and describe behaviors, we quantified activities performed for a period of time, and the number of activities or positions. We classified behaviors as states or events following Martin et al. [31]: an event corresponds to a behavior of short time duration, such as body movements, and a state corresponds to a behavior performed during a longer period of time, such as specific activities or postures. We performed observations of males and females under three conditions: solitary observations, conspecific interactions, and solitary facing a mirror. All behavioral observations and experiments were carried out during the light period without modifying other environmental conditions.

Solitary observations. We recorded isolated lizards inside their terrariums from 08:00 to 17:00 using a video camera Sony HDR-PJ430VE positioned vertically above the terrarium. Lizards were isolated in new terrariums, never occupied by another conspecific, for two weeks before recording. We recorded 12 lizards, 6 females and 6 males, between November and December 2016.

Conspecific interaction experiments. Lizards were randomly designated as focal animal (observed lizard) or stimulus animal (individuals used to elicit the focal lizard’s reaction, which were not filmed). The experiments were carried out during the light period between 9:00 and 11:00 am. Responses of focal lizards were filmed, from short distance (~30 cm), with the video camera accompanied by an observer. Two individuals were used per trial to test the behavioral responses of a focal animal caused by an animal of the same or opposite sex following two protocols. For lizards captured in 2016 (2016 experiments), we measured the response of a focal lizard to a stimulus created by a strange ‘stimulus lizard’ which was placed inside the enclosure of the focal lizard. For these experiments, focal animals were allowed to adjust to the terrarium alone for at least 48 h before the experiment, and only the focal lizard was filmed. Twelve same-sex (six male–male and six female–female) and six opposite-sex trials (three male–females and three female–male) were performed during January and February 2017. For lizards captured in 2017 (2017 experiments), we used a neutral glass observation chamber (80 cm × 50 cm) bisected by a removable opaque partition, fitted with a replaceable cardboard and not previously occupied by either individual. The chamber was washed, and the cardboard was replaced prior to each trial to eliminate potential chemical cues from previous occupants. The chamber walls were also covered with cardboard to prevent any visual disturbance, except the side of the observer. In these experiments, two lizards—the focal and stimulus individuals—were moved from their terrariums and placed at the same time at opposite ends of the chamber. After a 15-min acclimation period for both individuals, the plastic division was removed, and the lizards were allowed to interact. Only the focal lizard was filmed. For all experiments, focal lizards were never used more than once in same-sex or opposite-sex experiment, and a period of 48 h isolation was allowed before each stimulus lizard was used again. Interactions where halted when lizards moved away from each other, or when prolonged aggression occurred (e.g., biting). A total of 24 experiments were performed in the neutral glass observation chamber between September and December of 2017.

Solitary facing a mirror. We located individuals within a small cardboard box (30 cm × 30 cm × 20 cm) with a small mirror (10 cm × 7 cm) fixed to one of the walls. Two video cameras were set up using tripods, pointing towards the mirror from different angles within the cardboard. The box was discarded and the mirror washed after every experiment. We used six males and six females, and recorded 12 videos of the first responses of the focal lizard. For this experiment we only used lizards collected in 2017. 

### 2.3. Video Analysis

To analyze postures and movements, we quantified the behaviors using the software Boris v.7.0.10 [32]. This software allowed us to register the number of events and the duration of states occurring during the observation. Based on recurrent behaviors observed in all videos, we created an ethogram. 

In solitary observations, to quantify and compare exploratory behaviors we calculated the percentage of time spent moving of each individual (PTM) by quantifying the amount of time a lizard spent actively moving divided by the total amount of time spent outside its burrow (TTO). We tested for differences in PTM between males and females using the Wilcoxon Rank-Sum Test. For differences in basking time we used a *t*-test with paired samples.

For the 2016 and 2017 experiments, we focused on behaviors that imply a response from the opponent. To detect the similarity on behavioral responses of individuals in these different experiments, we built a similarity matrix using the Jaccard Index and the nearest neighbor cluster method, taking into consideration the presence/absence of behaviors during each experiment. For this analysis, we excluded behavioral observations which had less than three repetitions or occurred only in one occasion. We also performed a Kruskal Wallis test to compare the duration of interactions between conspecific experiments (male–male, female–female, male–females, and female–male). Statistical analyses and graphs were performed using RStudio V.1.3.1056 [33], with the packages Vegan [34], ggplot2 [35], and superheat [36].

## 3. Results

### 3.1. Ethogram

We observed thirty-four behaviors performed by *P. montium* in captivity (Table 1). Behaviors were grouped into three functional categories: maintenance (13), general locomotor patterns (4), conspecific elicited locomotor patterns (5), and conspecific elicited positions and movements (12). Maintenance represents actions that are normal for the species and are performed in solitary. General locomotor patterns correspond to unaccompanied displacement activities. Conspecific elicited patterns are displacement motivated by another lizard, and conspecific elicited positions and movements are behaviors that provide evidence of the animal’s motivation. Such individual behaviors are not necessarily exclusive to one category (Table 1). All behaviors listed as maintenance were observed in all individuals (male and female) during the focal solitary observations, mirror treatments or conspecific stimulus experiments.

### 3.2. Focal Solitary Observation and Measurement of Activity during the Day

**Figure 1:**
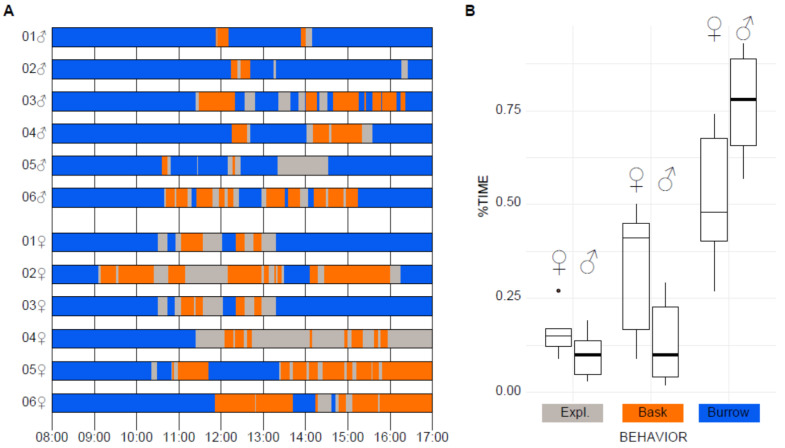
Exploratory (Expl.) behaviors in isolated lizards. (**A**) Scheme with the Distribution of Exploring (Gray), basking (Orange) and burrowing (Blue) behaviors across 9 h of observation for 12 individuals, 6 males and 6 females. (**B**) Boxplot for the percentage of time allocated to foraging (For), basking (Bask), and burrowing (Burrow) in males vs. females from Table 2. The boxes present a median line and standard deviation whiskers.

To assess reptile behavioral welfare in a laboratory experimental setup and to discover daily patterns of behaviors in isolated lizards, we documented approximately 108 h of focal solitary observations from six males and six females in their isolation terrariums (Figure 1A).

We classified observed behaviors as Exploratory (active behaviors), Basking, and Burrowing. Exploratory periods were characterized by displacement in the enclosure while moving the head side to side (looking around), protruding the tongue (tongue-flick), and capturing preys (eat) by digging (dig) to uncover crickets. Basking is the flattening of the ventral surface onto the substrate while the lizard is immobile. During burrowing periods, it was common to observe lizards hidden inside their burrows or under the water plate (burrow) or hidden under the substrate (bury; Table 1).

Exploratory behavior and basking outside burrow sites occurred towards noon, while burrowing commonly occurred during early hours or towards the end of the day (Figure 1A). Exploratory behaviors represented approximately 10% of daily activities in both, male and females (Figure 1B). The active periods were interspaced with inactive basking and burrowing behaviors (Figure 1A). We found no differences on the percentage of active time between males and females (Figure 1B; Table 2; Wilcoxon rank-sum Test W = 25.5, *p* = 0.2607). Basking was the most common single behavior recorded in inactive individuals outside its burrow, particularly in females; however, no significant difference was found on the percentage of basking time (t(6) = 2.1156, *p* = 0.088). We also found a slightly increased tendency of females to remain outside their burrow in comparison to males (TTO, Table 2, W = 30, *p* = 0.0259). However, there were no differences (W = 13, *p* = 0.4704) in the percentage of time in movement (PTM) between male (PTM♂ = 46.88 ± 6.48 S.E., Standard Error of the mean) and females (PTM♀ = 38.1 ± 5.86 S.E., Table 2).

### 3.3. Conspecific Interactions

**Figure 2:**
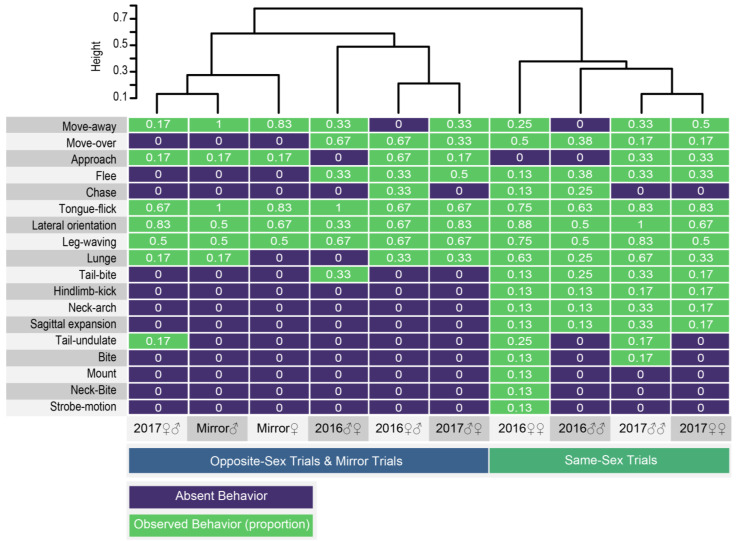
Mirror experiment responses are similar to opposite sex stimulus experiments. Heatmap from a presence–absence matrix of behaviors recorded in 10 conspecific stimulus experiments. Social locomotor patterns, positions, and movements are in the left. The dendrogram clustering is based on Jaccard distances from the similarity indexes on Supplementary Information 2. The numbers in white are the observed frequencies of each behavior and trial from Supplementary Information 1.

As we did not find stress-related behavior or abnormal interactions with the terrarium in isolated lizards, we designed two types of experiments to detect abnormal aggression patterns (territoriality) prompted by the owner of the area (2016 experiments) and to assess conspecific interactions without the influence of territoriality (2017 experiments; see methods) taking into account the sex of the focal lizard. In addition, we designed a mirror experiment to assess the response to a similar conspecific and guarantee the exclusive response to visual cues. In all these experiments, we identified 13 behavioral positions and movements, and five patterns of locomotion associated to the presence of a conspecific, or its reflection, in a controlled experiment (Table 1). To discover patterns of similarity among experiments and among responses of the focal lizard, we generated a heatmap from a matrix of the observed behaviors and its frequencies (Figure 2; Table S1). According to a clustering method based on Jaccard distances of a presence–absence matrix (Table S2), there are two distinctive groups: (1) the focal stimulus experiments with opposite-sex interaction are similar to mirror experiments, and (2) the focal stimulus experiments with same-sex interaction are similar between them regardless the experimental setup (2016 experiments vs. 2017 experiments), and of the sex of the focal lizard (female–female and male–male) (Figure 2). The duration of each interaction varied depending on the response of the contenders but was not significantly different between experiments (H = 3.83, *p* = 0.28).

**Figure 3:**
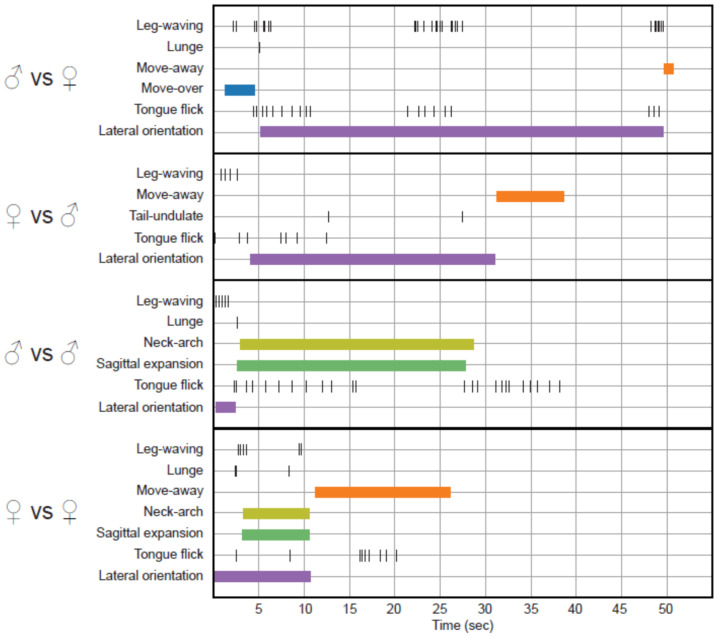
Time budget graphic in four experiments, one per stimulus context. We selected interactions which presented the largest number of performed behaviors.

Most conspecific elicited locomotor patterns and the movements, like Tongue-flick, Lateral orientation, Leg-waving, and Lunge, were observed in both stimulus treatments. Lateral orientation, tongue-flick, and Leg-waving are the most common movements, and tongue-flick is persistent during most interactions (Figure 3). These movements together with Neck-arch were the first to occur chronologically in a treatment in response to the presence of a conspecific (Figure 3).

Among conspecific elicited positions and movements, nine behaviors were observed almost exclusively in same sex interactions: Tail-bite, Hindlimb-kick, Neck-arch, Sagittal expansion, Tail-undulate, Bite, Mount, Neck-Bite, and Strobe-motion.

**Figure 4:**
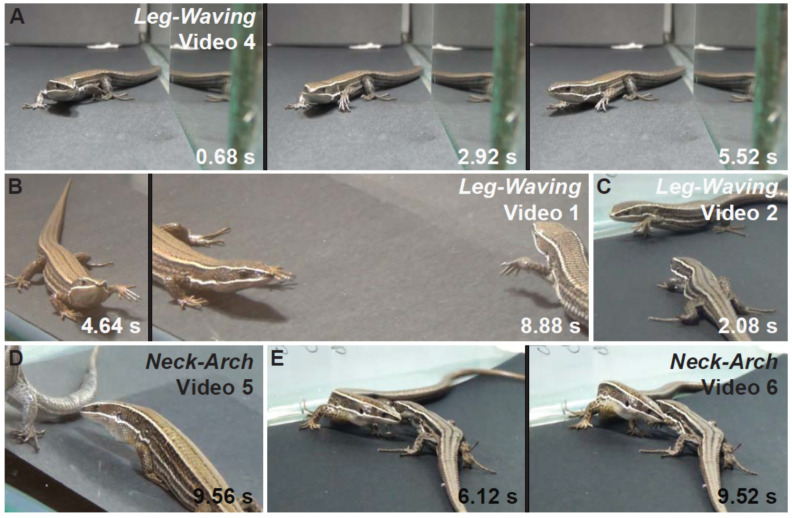
Leg-waving and neck-arch behaviors in *P. montium*. Still images and series from the Supplementary Videos. The time in the lower corner is in seconds and fractions of seconds (0.00 s). (**A**) Leg-waving in a mirror experiment performed by a Female (Video S4). (**B**,**C**) Mutual leg-waving performed by two males. (**D**,**E**) Neck-arch behavior performed by two males. Video S6 is a continuation from Video S2.

Outstandingly, we observed two movements, leg-waving and neck-arch (Figure 3 and Figure 4), which were expressed at a distance from the receiver; they occur pre-contact and did not necessarily require physical contact to be elicited. From these two, only leg-waving was observed in mirror treatments.

Leg-waving in *P. montium* consists of the complete elevation of one of the forelegs, followed by swinging of the humerus locked with the radius-ulna in the dorso-ventral plane (Figure 4A–C; Videos S1–S4). In some cases, the hand also produced an independent swinging in the same plane, concomitant to the limb movement (Videos S3 and S4). This complex movement is often integrated to the locomotion of the lizard: when the waving limb is lifted, the propulsive strokes of the other limbs start, then the whole body is pushed forward and maintained in elevation while the forelimb waves. Finally, the waving limb will fall in a more anterior position to where it was lifted, completing a single step (Videos S1–S3). We also observed leg waving in static lizards (Video S4). Leg-waving was usually accompanied or even elicited by head movements and tongue flicking and occurred only when the lizard showed lateral orientation to the stimulus. The speed and number of limb movements, and forelimb used (left vs. right), varied between individuals (Figure 3); however, the leg used for this movement was always the one directed towards the contender. From our observations, the number of males and females performing this behavior was very similar, and there was no remarkable difference in use of this movement in other conspecific interactions (Figure 2).

Neck-arch consists of the rising of the lizard’s body and the stretching of its front legs while the snout is pointed towards the ground. The movement is always accompanied by sagittal expansion, which is the lateral compression of the body producing an enlargement along the sagittal plane (Figure 4D,E; Videos S5 and S6). This was observed in a few occasions, only in same sex treatments (Figure 2). This behavior does not imply the lack of body motion as we registered two males walking while maintaining this posture (Figure 4E, Videos S5 and S6). In some treatments, neck-arch was performed while the two contenders stood parallel to each other facing opposite directions, in mutual lateral orientation (Figure 4E). We observed a peculiar position in two males, one front leg raised off the ground and held against the side of the body while neck-arching. Interestingly, in three out of five experiments, neck-arch was followed by leg-waving and antagonistic tail biting (Figure 4; Video S6).

## 4. Discussion

### 4.1. Visual Signaling in Gymnophtalmidae

Despite being one of the most diverse families in the Neotropical region, with more than 250 species [37,38], we know very little regarding behavior and communication in gymnophthalmid lizards, and in most fossorial or semi-fossorial squamates [11]. This is probably because of its reduced size and cryptic lifestyle. Through ex situ observation, we produced the first detailed ethogram of a gymnophthalmid lizard including information regarding activity patterns, and the first evidence in this family of visual signaling used under conspecific-interaction experiments.

Our behavioral analyses of the gymnophthalmid lizard *P. montium*, although preliminary due to the limited number of lizards, show a complex repertoire of movement-based behaviors. During these observations, tongue flicking was the most common movement performed in every experiment (Figure 3), indicating a possibly strong tendency to vomerolfaction [39] that still needs to be studied.

In addition, we were able to observe stimuli-elicited movements like leg waving, neck arching, and tail undulation that were not produced in solitary observations and may have potential roles as visual signals during social interactions. So far, the only gymnophthalmid on which a stimulus elicited movement has been previously recorded is *Calyptommatus leiolepis*, a fossorial legless lizard that produces intense tail movements during mating [40]. It is not easy to classify tail movements as visual signaling, as it could be considered an involuntary movement provoked by mating or predatory stress. For instance, it is hypothesized that tail undulation could be associated to tail autotomy, as waving the tail may direct the predator’s attention towards its more “expendable” appendage [41,42]. Other movements related to tail undulate, like tail lashing, tail coiling, and tail waving are often recorded as intraspecific territorial, aggressive, or deterrent behaviors in Agamids, Iguanids, and Lacertids [43,44,45,46]. Tail undulation in *P. montium* was also recorded in our experiments, but only in same-sex interactions and in such a low frequency that does not allow analyzing its potential as a visual signal.

From the conspecific elicited movements recorded in *P. montium*, we highlight leg-waving and neck-arching as the most relevant visual signals since they were elicited at a distance from the stimulus individual. A stronger case can be made for leg-waving, as it was observed in every conspecific stimulus experiment and in the mirror experiments. Signals that involve limb movement like raising, waving, or circumduction, have never been reported in Gymnophthalmidae. However, these signals are common among iguanian lizards, particularly in the Agamidae family [3,4,11]. Leg waving has also been described in Lacertidae, Scincidae, and Teiidae [47,48,49]. In the Bonaire whiptail lizard *Cnemidophorus murinus* (Teiidae), leg movements described as circumduction or waving have been shown to be elicited on the presence of attackers or predators, and may have a deterrence function [47]. In *Podarcis muralis*, leg waving seems to be only used at a distance and to deter predators from chasing them [50]. During observations of isolated individuals, we never observed leg-waving elicited by the presence of any human observer, nor did we observe it when collecting lizards in the field. Considering that, in our experiments, *P. montium* individuals only performed this behavior in the presence of a conspecific, and contenders frequently responded by performing the same signal, it is possible that leg-waving plays a specific role in deterring against conspecific aggression or in conspecific recognition.

In the field, *P. montium* females and males spend most of their time hidden in burrows or basking nearby. As diurnal lizards with an apparent lack of territoriality, their foraging behavior might force them to become exposed to other conspecifics while searching for food or a nesting place [25]. We hypothesize *P. montium* could have evolved visual communication as a deterrence signal against conspecific aggression when competing for a burrow, resources, or mating. Nevertheless, conspecific aggression was not deterred in interactions that included leg-waving; therefore, leg-waving might have a distinct within a multimodal context (discussed below). Still, further experimentation such as analyzing the presence of a predator or an attacker’s simulation might help to confirm the role of leg-waving or neck-arching as a deterrence signal in *P. montium*. Predation deterrence also needs to be tested both in laboratory and in situ.

Regarding neck-arching, this behavior was performed during conspecific stimulus experiments both with same-sex and opposite sex stimulus lizards; therefore, a deterrence role can also be presumed. Neck-arching is considered a dominant behavior or a challenge behavior by appearing larger than the contender as shown in several scincids like *Lamproholis guichenoti* [51]. This visual signal is also related to the exhibition of body color patterns such as spots or rows, which reveal information regarding individuals fitness or fighting abilities [52]. During *P. montium* interactions, neck-arching allowed the exposition of the lateral colored bands characteristics of the species; nonetheless, nothing is known regarding the ability of the Andean lizard to detect colored patches. There are 14 species currently placed in this genus, some of which have bright color patterns, and similar semi-fossorial adaptations [21,37].

### 4.2. Multimodal Signaling in Lizards

Some behaviors observed in *P. montium* are similar to lizards of the Teiidae family, the sister clade of Gymnophthalmidae, and to some non-related lizards like iguanids and lacertids [11,42,48,53,54]. This confirms that visual communication and multimodal signaling, or the combinatorial usage of more than one communication modality, are widespread across Squamata. Still, multimodal signaling has only been studied in species with conspicuous visual signaling [1,6,11,16]. For groups like Teiidae and Scincidae, which were commonly thought to rely heavily on chemosensation, only a few context-dependent visual displays have been described [11].

The adaptive value or advantage of using multiple signaling modalities could be reinforcement of a single message or the diversification of messages that an individual could provide [5,55]. From the analysis of visual and chemical traits in geckos, it has been hypothesized that multimodal signaling could enhance message delivery when other constrains negatively affects the intensity of one of the signals [56]. As previously mentioned, *P. montium* lacks femoral follicular glands [21,23,25]. Therefore, we could hypothesize that multimodality in this species is the result of signal diversification to compensate the reduction of chemical signal sources.

Still, leg-waving did not deter intrasexual aggression in *P. montium*, suggesting that leg-waving might not be used for attack deterrence but for delivering a different message. When analyzed separately, it is generally thought that chemical signaling is dominant in sexual interactions [16,17], while visual signals provide a deterrent or conspecific recognition message, as discussed above. Intrasexual aggression, particularly, has been shown to be guided mostly by pheromonal signals in *Anolis* and *Podarcis* lizards, which also use visual signals to communicate [57,58]. In our results, the lack of aggressive behavior and use of leg-waving in mirror assays (Figure 2) supports the idea of message diversification in *P. montium*. However, the limited number of sampled individuals, the limitations in the experimental design and confounding effects in the assays, only allow us to speculate regarding the biological relevance of these observations. Further experimentation is required to determine other potential roles of leg waving and the advantages of multimodal communication in *P. montium*.

### 4.3. Ex Situ Behavioral Studies in Pholidobolus Montium

As we have shown, the observation of lizards in a laboratory setting is one possible approach to study the modalities of communication in gymnopthalmid lizards, and other fossorial or semi-fossorial lizards. Through this approach, we were able to witness conspecific elicited behaviors that would not have been possible to record in situ. On the other hand, the observed behaviors could be associated to captivity-induced stress. An excess of repetitive behaviors, the excessive interaction with the walls of the enclosure, or the depression in the daily pattern of exploratory movements of a diurnal lizard have been described as a consequence of acute stressors [59]. To address potential maladaptive behavior, we recorded behavior of isolated lizards for more than 9 hrs. and concluded that *P. montium* individuals (1) retain their diurnal behavior in captivity (Figure 1A), (2) do not show stress-associated movements, and (3) maintain an “average” foraging behavior. Foraging in *P. montium* was assessed using the observed percentage of time in movement (PTM; Figure 1B, Table 1) [60]. The PTM exhibited by *P. montium* isolated individuals (Table 2. PTM = 42.49 ±6.32 S.E) falls within the range of an active foraging strategy and could be comparable to the PTM that some scincid or lacertid species show in the wild [60,61]. In addition, we did not register reactive hiding in the presence of an observer, excessive interaction with the terrarium glass or other behavioral pattern associated with stress in reptiles [62,63]. Furthermore, we did not record color changes in adults during their time in captivity or drastic weight loss (results not shown). These evidences may indicate that the artificial enclosure or laboratory settings were potentially appropriate for individual dwelling and mating purposes.

Maladaptive behaviors could also develop due to undetected stress on individuals in captivity. In our experiments, we noted that *P. montium* same-sex interactions presented a wider variety of behavioral responses compared to opposite-sex interactions. Most of the same-sex “exclusive” behaviors seem to be antagonistic (Figure 2; Neck-arch, tail-bite, neck-bite, hidlimb-kick). Neck-arching and sagittal-expansion have been previously described as threatening reactions in male–male interactions in *Varanus gouldii* [64], *Anolis proboscis* [65], and *Uta stansburiana* [66]. In *P. montium,* we observed neck-arching displays during antagonistic interactions male–male and female–female, followed by aggressive behaviors like tail-bite and hindlimb-kick, which suggest a threatening function. The aggressive behavior observed in our conspecific stimulus experiments could be an artifact related to the unnatural environment of the empty tank used for these experiments that might have caused the sensation of depleted resources. Indeed, female–female aggression in lizards has been recently linked to resource competition in the agamid lizard *Phrynocephalus vlangalii* [67]. Still, the differences in aggressiveness observed in same-sex compared to opposite-sex laboratory treatments might have complex multimodal basis that should be further studied. Even though behavioral analysis like this can be used to assess lizard welfare and stress in captivity, further analyses of stress relevant hormones in circulating blood should also be made.

Unfortunately, *P. montium* adaptation to urban areas, where these lizards were commonly observed until the end of last century [26], is also the main reason why *P. montium* is currently a near threatened species. This is particularly true in urban areas where populations have been decimated possibly due to predation by invasive species and pets [29]. Therefore, further studies regarding *P. montium* natural history are sorely needed in order to increase the awareness regarding the current threats against this species and to propose potential ex situ management projects.

## 5. Conclusions

Our observations contribute to understand the communication modalities used in semi-fossorial lizards. We demonstrate that *Pholidobolus montium* can use visual signals to communicate. This could be an evidence of multimodality as this species also present tongue flicking movements with a potential chemical sampling role. Leg-waving and neck-arching might be used by *P. montium* as a deterrence signal; however, further studies are still needed.

## Figures and Tables

**Table 1:** Ethogram of *Pholidobolus montium.* Behaviors of individuals maintained in captivity are listed and have been assigned to functional categories based on current observations and previous studies. The symbols imply states (~) or events (*).

Functional Category	Behavior	Description	Occur during Solitary obs.	Occur during Conspecific Stimulus Experiments	Occur during Mirror Experiments
Maintenance					
	Adpress *	One or more limbs are raised off the substrate and held against the side of the body.	yes	no	no
	Bask ~	Dorsoventral flattening of the body onto the substrate.	yes	no	no
	Thread *	Lizard rolls its body sideways.	yes	no	no
	Burrow ~	Lizard hides inside its burrow.	yes	no	no
	Bury ~	Hide underground by digging down the substrate.	yes	no	no
	Cloacal-drag ~	Full body displacement frontward while keeping the cloacal region in contact to the substrate.	yes	no	no
	Dig ~	Lizard removes the substrate with its forelimbs.	yes	no	no
	Drink ~	Snout placed into the water and tongue is slowly protruded and returned to the mouth.	yes	no	no
	Eat ~	A food item is grasped with the jaws and ingested.	yes	no	no
	Tongue-flick *	Tongue is protruded and returned to the mouth.	yes	yes	yes
	Scratch *	Hind limb movements used to rapidly scrape the body bowed laterally.	yes	no	no
	Slough *	Scrapes its body against stationary objects as to remove sloughed skin.	yes	no	no
	Looking-around ~	Side to side movement of the head, while the body remains motionless.	yes	no	no
General Locomotor patterns					
	Walk ~	Forward movement with the ventral region of the body in contact with the substrate.	yes	yes	yes
	Jump ~	Fast leap, all four feet are not in contact to the substrate.	yes	no	no
	Run ~	Fast forward movement with the body raised off the substrate.	yes	yes	yes
	Stalk ~	Slow walking movement.	yes	yes	yes
Conspecific elicited locomotor patterns					
	Approach ~	One lizard moves toward another up to a considerable distance, which does not allow physical interaction.	no	yes	yes
	Chase ~	Rapid pursuit of one lizard to another.	no	yes	no
	Flee *	Rapid evacuation when chased by another individual.	no	yes	no
	Move-away ~	One lizard slowly passes over another.	no	yes	yes
	Move-over ~	One lizard moves over the top of another.	no	yes	no
Conspecific elicited positions and movements					
	Bite *	Grip another individual with its jaws.	no	yes	no
	Hindlimb-kick *	Push away another individual by using one hindlimb.	no	yes	no
	Lateral orientation ~	Face another individual laterally, the sagittal plane of the entire body or, at least the anterior part, is presented.	no	yes	yes
	Leg-waving *	Lizard elevates and swings a foreleg.	no	yes	yes
	Lunge *	One lizard rapidly moves toward and away from another.	no	yes	yes
	Mount *	One lizard steps/stands on the antagonist’s dorsum.	no	yes	no
	Neck-arch ~	Lizard raises its body using push up (stretch front legs), while the snout is pointed toward the ground.	no	yes	no
	Neck-bite *	Lizard bits the skin on the neck of another.	no	yes	no
	Sagittal expansion ~	Lateral compression of the body and dorsoventral expansion.	no	yes	no
	Strobe-motion *	Short rapid jerks.	no	yes	no
	Tail-bite *	Grasp another lizard’s tail in its jaws.	no	yes	no
	Tail-undulate *	A sinusoidal, horizontal movement of the entire tail.	no	yes	yes

**Table 2:** Percentage of time used by males (M) or Females (F) during focal solitary observations. The total observation time is 9 h for each individual. The Exploratory and Inactive categories involve multiple behaviors enlisted in Table 1. S.E. is the Standard Error of the Mean. PTM. Percentage of time in movement. TTO. Total time outside.

Individual	Exploratory	Basking	Hiding /Burrowing	PTM	TTO (min)
M1	2.81	4.08	93.11	40.76	37.21
M2	4.14	3.99	91.91	50.94	43.65
M3	12.73	24.97	62.36	33.77	203.6
M4	7.06	15.72	77.23	30.98	123.01
M5	19.5	1.58	78.94	92.5	113.81
M6	13.91	29.13	57	32.32	232.43
$\bar{X}$ _M_	10.02	13.25	76.76	46.88	125.62
S.E.	1.77	3.26	4.08	6.48	22.08
F1	16.57	9.36	74.09	63.91	139.99
F2	27.49	45.87	26.69	37.47	396.11
F3	16.87	9.36	73.86	64.33	141.64
F4	12.19	51.34	38.2	19.18	343.04
F5	14.14	40.81	47.68	25.73	296.7
F6	9.13	41.65	49.24	17.98	274.18
$\bar{X}$ _F_	16.06	33.06	51.63	38.1	265.28
S.E.	1.73	5.16	5.25	5.86	28.92
$\bar{X}$	13.04	23.15	64.19	42.49	195.45
S.E.	1.98	5.25	6.04	6.32	33.25

## Data Availability

Raw data available at: https://figshare.com/projects/Poma_et_al_2021_RAW_DATA_Animals_sub/121371. Published on 24 August 2021.

## Supplementary Materials

The following are available online at https://www.mdpi.com/article/10.3390/ani11113022/s1, Table S1. Proportion of individuals seen performing a behavior in each conspecific stimulus context. Table S2. Similarity matrix with Jaccard distances calculated from the presence-absence of a behavior in Table S1. Video S1—Leg waving couple M12 vs. M18. Video S2—Leg waving couple M09 vs. M03. Video S3—Leg waving mirror M18. Video S4—Leg waving mirror F05. Video S5—Neck Arching M09 vs. M03. Video S6—Leg waving Neck Arching couple M09 vs. M03.

## References

[1] Baeckens S., Driessens T., Van Damme R. (2016). Intersexual chemo-sensation in a “visually-oriented” lizard, *Anolis sagrei*. PeerJ.

[2] Martins E.P., Bissell A.N., Morgan K.K. (1998). Population differences in a lizard communicative display: Evidence for rapid change in structure and function. Anim. Behav..

[3] Radder R.S., Saidapur S.K., Shine R., Shanbhag B.A. (2006). The language of lizards: Interpreting the function of visual displays of the Indian rock lizard, *Psammophilus dorsalis* (Agamidae). J. Ethol..

[4] Ramos J.A., Peters R.A. (2016). Dragon wars: Movement-based signalling by Australian agamid lizards in relation to species ecology. Austral Ecol..

[5] Fleishman L.J., Font E., Bels V.L., Russell A.P. (2019). Sensory Processing in Relation to Signaling Behavior. Behavior of Lizards.

[6] Marcellini D. (1977). Acoustic and visual display behavior of gekkonid lizards. Integr. Comp. Biol..

[7] Fleishman L.J., Ogas B., Steinberg D., Leal M. (2016). Why do *Anolis* dewlaps glow? An analysis of a translucent visual signal. Funct. Ecol..

[8] Mason R.T., Gutzke W.H.N. (1990). Sex recognition in the leopard gecko, *Eublepharis macularius* (Sauria: Gekkonidae) Possible mediation by skin-derived semiochemicals. J. Chem. Ecol..

[9] Mayerl C., Van Damme R., Baeckens S. (2015). Evolution and role of the follicular epidermal gland system in non-ophidian squamates. Amphib. Reptil..

[10] Moreira P.L., López P., Martín J. (2008). Discrimination of conspecific faecal chemicals and spatial decisions in juvenile Iberian rock lizards (*Lacerta monticola*). Acta Ethol..

[11] Johnson M.A., Cook E.G., Kircher B.K., Bels V.L., Russell A.P. (2019). Phylogeny and Ontogeny of Display Behavior. Behavior of Lizards.

[12] Burghardt G.M. (1980). Behavioral and Stimulus Correlates of Vomeronasal Functioning in Reptiles: Feeding, Grouping, Sex, and Tongue Use. Chemical Signals.

[13] Cooper W.E. (1995). Foraging mode, prey chemical discrimination, and phylogeny in lizards. Anim. Behav..

[14] Schwenk K. (1995). Of tongues and noses: Chemoreception in lizards and snakes. Trends Ecol. Evol..

[15] Vitt L.J., Pianka E.R., Cooper W.E., Schwenk K. (2003). History and the Global Ecology of Squamate Reptiles. Am. Nat..

[16] García-Roa R., Jara M., Baeckens S., López P., Van Damme R., Martín J., Pincheira-Donoso D. (2017). Macroevolutionary diversification of glands for chemical communication in squamate reptiles. Sci. Rep..

[17] García-Roa R., Jara M., López P., Martín J., Pincheira-Donoso D. (2017). Heterogeneous tempo and mode of evolutionary diversification of compounds in lizard chemical signals. Ecol. Evol..

[18] Schwenk K. (1993). The Evolution of Chemoreception in Squamate Reptiles: A Phylogenetic Approach. Brain Behav. Evol..

[19] Teixeira M., Recoder R.S., Camacho A., De Sena M.A., Navas C.A., Rodrigues M.T. (2013). A new species of *Bachia* Gray, 1845 (Squamata: Gymnophthalmidae) from the Eastern Brazilian Cerrado, and data on its ecology, physiology and behavior. Zootaxa.

[20] Goicoechea N., Frost D.R., De la Riva I., Pellegrino K.C.M., Sites J., Rodrigues M.T., Padial J.M. (2016). Molecular systematics of teioid lizards (Teioidea/Gymnophthalmoidea: Squamata) based on the analysis of 48 loci under tree-alignment and similarity-alignment. Cladistics.

[21] Parra V., Nunes P.M.S., Torres-Carvajal O. (2020). Systematics of *Pholidobolus* lizards (Squamata, Gymnophthalmidae) from southern Ecuador, with descriptions of four new species. Zookeys.

[22] Peters W.C.H. (1863). Über Cercosaura und Die Mit Dieser Gattung verwandten Eidechsen aus Südamerica.

[23] Hillis D.M. (1985). Evolutionary Genetics of The Andean Lizard Genus *Pholidobolus* (Sauria: Gymnophthalmidae): Phylogeny, Biogeography, And Comparison Of Tree Construction Techniques. Syst. Biol..

[24] Dávila-Jativa M., Cisneros-Heredia D. (2017). Use of human-made buildings by *Stenocercus* lizards (Iguania, Tropiduridae). Herpetol. Notes.

[25] Montanucci R.R. (1973). Systematics and evolution of the andean lizard genus *Pholidobolus* (Sauria: Teiidae). Univ. Kansas Museum Nat. Hist. Misc. Publ..

[26] Ramírez-Jaramillo S. (2016). Nidos de *Pholidobolus montium* en una área intervenida de Mulaló, Cotopaxi-Ecuador. Rev. Ecuat. Med. Cienc. Biol..

[27] Bustard R. (1964). Egg laying and incubation of the striped mountain lizard *Pholidobolus montium* (Teiidae) with notes on an incubator. Br. J. Herpetol..

[28] Doody J.S., Freedberg S., Keogh J.S. (2009). Communal egg-laying in reptiles and amphibians: Evolutionary patterns and hypotheses. Q. Rev. Biol..

[29] Cisneros-Heredia D.F. Pholidobolus montium (Errata Version Published in 2017). https://www.iucnredlist.org/species/44578680/115386433.

[30] ASIH Guidelines for Live Amphibians and Reptiles in Field and Laboratory Research. https://asih.org/sites/default/files/2018-05/guidelines_herps_research_2004.pdf.

[31] Martin P., Bateson P.P.G. (1993). Measuring Behaviour: An Introductory Guide.

[32] Friard O., Gamba M. (2016). BORIS: A free, versatile open-source event-logging software for video/audio coding and live observations. Methods Ecol. Evol..

[33] RStudio Team (2020). RStudio: Integrated Development for R.

[34] Oksanen J.F., Blanchet G., Friendly M., Kindt R., Legendre P., McGlinn D., Minchin P.R., O’Hara R.B., Simpson G.L., Solymos P. (2019). Vegan: Community Ecology Package, R Package Version 2.5-6. https://cran.microsoft.com/snapshot/2019-12-24/web/packages/vegan/index.html.

[35] Wickham H. (2016). ggplot2: Elegant Graphics for Data Analysis.

[36] Barter R.L., Yu B. (2018). Superheat: An R package for creating beautiful and extendable heatmaps for visualizing complex data. J. Comput. Graph. Stat..

[37] Torres-Carvajal O., Lobos S.E., Venegas P.J., Chávez G., Aguirre-Peñafiel V., Zurita D., Echevarría L.Y. (2016). Phylogeny and biogeography of the most diverse clade of South American gymnophthalmid lizards (Squamata, Gymnophthalmidae, Cercosaurinae). Mol. Phylogenet. Evol..

[38] Uetz P., Etzold T. (1996). The EMBL/EBI Reptile Database. Herpetol. Rev..

[39] Cooper W.E. (1994). Chemical discrimination by tongue-flicking in lizards: A review with hypotheses on its origin and its ecological and phylogenetic relationships. J. Chem. Ecol..

[40] Duran Filho C., Molina F.B. (2002). O Comportamento de Acasalamento de *Calyptommatus leiolepis* Rdorigues, 1992 em Cativerio (Sauria, Gymnophthalmidae: Observacoes Preliminares. Rev. Etol..

[41] Clause A., Capaldi E. (2006). Caudal Autotomy and Regeneration in Lizards. J. Exp. Zool..

[42] Torr G.A., Shine R. (1994). An ethogram for the small scincid lizard *Lampropholis guichenoti*. Amphibia-Reptilia.

[43] Van Dyk D.A., Evans C.S. (2008). Opponent assessment in lizards: Examining the effect of aggressive and submissive signals. Behav. Ecol..

[44] Font E., Barbosa D., Sampedro C., Carazo P. (2012). Social behavior, chemical communication, and adult neurogenesis: Studies of scent mark function in *Podarcis* wall lizards. Gen. Comp. Endocrinol..

[45] Ord T.J., Peters R.A., Evans C.S., Taylor A.J. (2002). Digital video playback and visual communication in lizards. Anim. Behav..

[46] Peters R.A., Ramos J.A., Hernandez J., Wu Y., Qi Y. (2016). Social context affects tail displays by *Phrynocephalus vlangalii* lizards from China. Nat. Publ. Gr..

[47] Cooper W.E., Pérez-Mellado V., Baird T.A., Caldwell J.P., Vitt L.J. (2004). Pursuit deterrent signalling by the bonaire whiptail lizard *Cnemidophorus murinus*. Behaviour.

[48] Langkilde T., Schwarzkopf L., Alford R. (2003). An ethogram for adult male rainbow skinks, *Carlia jarnoldae*. Herpetol. J..

[49] Carpenter C.C., Ferguson G.W. (1977). Variation and evolution of stereotyped displays in reptiles. Biol. Reptil. Ecol. Behav..

[50] Font E., Carazo P., Pérez i de Lanuza G., Kramer M. (2012). Predator-elicited foot shakes in Wall Lizards (*Podarcis muralis*): Evidence for a Pursuit-Deterrent function. J. Comp. Psychol..

[51] Torr G.A., Shine R. (1996). Patterns of Dominance in the Small Scincid Lizard *Lampropholis guichenoti*. J. Herpetol..

[52] Bro-Jørgensen J. (2010). Dynamics of multiple signalling systems: Animal communication in a world in flux. Trends Ecol. Evol..

[53] Greenberg N. (1977). An Ethogram of the Blue Spiny Lizard, *Sceloporus cyanogenys* (Reptilia, Lacertilia, Iguanidae). J. Herpetol..

[54] Qi Y., Li S., Suo L., Li H., Wang Y. (2011). An ethogram of the toad-headed lizard *Phrynocephalus vlangalii* during the breeding season. Asian Herpetol. Res..

[55] Whiting M.J., Miles D.B., Bels V.L., Russell A.P. (2019). Behavioral Ecology of Aggressive Behavior in Lizards. Behavior of Lizards.

[56] Kabir M.S., Venkatesan R., Thaker M. (2020). Multiple sensory modalities in diurnal geckos is associated with the signalling environment and evolutionary constraints. Integr. Org. Biol..

[57] Reedy A.M., Pope B.D., Kiriazis N.M., Giordano C.L., Sams C.L., Warner D.A., Cox R.M. (2017). Female anoles display less but attack more quickly than males in response to territorial intrusions. Behav. Ecol..

[58] López P., Martín J., Cuadrado M. (2002). Pheromone-Mediated Intrasexual Aggression in Male Lizards, *Podarcis hispanicus*. Aggress. Behav..

[59] Martin J., Salvador A. (1997). Effects of Tail Loss on the Time-Budgets, Movements, and Spacing Patterns of Iberian Rock Lizards, Lacerta monticola. Herpetologica.

[60] Miles D.B., Losos J.B., Irschick D.J., Miles D.B., McBrayer L.B., Reilly S.M. (2007). Morphology, performance, and foraging mode. Lizard Ecology.

[61] Cooper W.E. (2005). The foraging mode controversy: Both continuous variation and clustering of foraging movements occur. J. Zool..

[62] Martínez Silvestre A. (2014). How to assess stress in reptiles. J. Exot. Pet Med..

[63] Warwick C., Arena P., Lindley S., Jessop M., Steedman C. (2013). Assessing reptile welfare using behavioural criteria. In Pract..

[64] Murphy J.B., Mitchell L.A. (1974). Ritualized Combat Behavior of the Pygmy Mulga Monitor Lizard, *Varanus gilleni* (Sauria: Varanidae). Herpetologica.

[65] Quirola D.R., Mármol A., Torres-Carvajal O., Narváez A.E., Ayala-Varela F., Moore I.T. (2017). Use of a rostral appendage during social interactions in the Ecuadorian *Anolis proboscis*. J. Nat. Hist..

[66] Brandt Y. (2003). Lizard threat display handicaps endurance. Proc. R. Soc. B Biol. Sci..

[67] Wu Y., Whiting M.J., Fu J., Qi Y. (2019). The driving forces behind female-female aggression and its fitness consequence in an Asian agamid lizard. Behav. Ecol. Sociobiol..

