# Supplementary material for: Visual Signaling in the Semi-Fossorial Lizard Pholidobolus montium (Gymnophthalmidae)
Source: Animals (Basel). 2021 Oct 21;11(11):3022. doi: 10.3390/ani11113022 (PMC8614464; doi:10.3390/ani11113022)
Supplement: Supplementary file 1 [file animals-11-03022-s001.zip › 20210802_Table S1.pdf]

Table S1. Proportion of individuals seen performing a behavior in each conspecific stimulus context. The* marks behaviors with possible function in visual communication. N is the number of experiments performed.

Behavior		Mirror		Opposite sex				Same sex			
	Year	2017	2017	2016	2016	2017	2017	2016	2016	2017	2017
	N	6♂	6♀	3♂♀	3♀♂	6♂♀	6♀♂	8♂♂	8♀♀	6♂♂	6♀♀
**Social locomotor patterns**											
Move-away		1.00	0.83	0.33	0.00	0.33	0.17	0.00	0.25	0.33	0.50
Move-over		0.00	0.00	0.67	0.67	0.33	0.00	0.38	0.50	0.17	0.17
Approach		0.17	0.17	0.00	0.67	0.17	0.17	0.00	0.00	0.33	0.33
Flee		0.00	0.00	0.33	0.33	0.50	0.00	0.38	0.13	0.33	0.33
Chase		0.00	0.00	0.00	0.33	0.00	0.00	0.25	0.13	0.00	0.00
**Social positions and movements**											
Tongue-flick		1.00	0.83	1.00	0.67	0.67	0.67	0.63	0.75	0.83	0.83
Lateral orientation		0.50	0.67	0.33	0.67	0.83	0.83	0.50	0.88	1.00	0.67
*Leg-waving**		0.50	0.50	0.67	0.67	0.67	0.50	0.50	0.75	0.83	0.50
Lunge		0.17	0.00	0.00	0.33	0.33	0.17	0.25	0.63	0.67	0.33
Tail-bite		0.00	0.00	0.33	0.00	0.00	0.00	0.25	0.13	0.33	0.17
Hindlimb-kick		0.00	0.00	0.00	0.00	0.00	0.00	0.13	0.13	0.17	0.17
*Neck-arch**		0.00	0.00	0.00	0.00	0.00	0.00	0.13	0.13	0.33	0.17
Sagittal expansion		0.00	0.00	0.00	0.00	0.00	0.00	0.13	0.13	0.33	0.17
*Tail-undulate**		0.00	0.00	0.00	0.00	0.00	0.17	0.00	0.25	0.17	0.00
Bite		0.00	0.00	0.00	0.00	0.00	0.00	0.00	0.13	0.17	0.00
Mount		0.00	0.00	0.00	0.00	0.00	0.00	0.00	0.13	0.00	0.00
Neck-Bite		0.00	0.00	0.00	0.00	0.00	0.00	0.00	0.13	0.00	0.00
Strobe-motion		0.00	0.00	0.00	0.00	0.00	0.00	0.00	0.13	0.00	0.00
