# Supplementary material for: Visual Signaling in the Semi-Fossorial Lizard Pholidobolus montium (Gymnophthalmidae)
Source: Animals (Basel). 2021 Oct 21;11(11):3022. doi: 10.3390/ani11113022 (PMC8614464; doi:10.3390/ani11113022)
Supplement: Supplementary file 1 [file animals-11-03022-s001.zip › 20210802_Table S2.pdf]

Table S2. Similarity matrix with Jaccard distances calculated from the presence-absence of a behavior in Table S1

			**Mirror**		**Opposite sex**				**Same sex**			
		Year	2017	2017	2016	2016	2017	2017	2016	2016	2017	2017
		N	6♂	6♀	3♂♀	3♀♂	6♂♀	6♀♂	8♂♂	8♀♀	6♂♂	6♀♀
**Mirror**	2017	6♂	0.000	0.286	0.444	0.333	0.125	0.250	0.615	0.667	0.500	0.417
	2017	6♀	0.286	0.000	0.500	0.556	0.375	0.286	0.769	0.778	0.643	0.583
**Opposite sex**	2016	3♂♀	0.444	0.500	0.000	0.500	0.333	0.600	0.500	0.588	0.500	0.417
	2016	3♀♂	0.333	0.556	0.500	0.000	0.222	0.500	0.417	0.611	0.533	0.462
	2017	6♂♀	0.125	0.375	0.333	0.222	0.000	0.333	0.538	0.611	0.429	0.333
	2017	6♀♂	0.250	0.286	0.600	0.500	0.333	0.000	0.714	0.667	0.500	0.538
**Same sex**	2016	8♂♂	0.615	0.769	0.500	0.417	0.538	0.714	0.000	0.353	0.333	0.231
	2016	8♀♀	0.667	0.778	0.588	0.611	0.611	0.667	0.353	0.000	0.278	0.389
	2017	6♂♂	0.500	0.643	0.500	0.533	0.429	0.500	0.333	0.278	0.000	0.143
	2017	6♀♀	0.417	0.583	0.417	0.462	0.333	0.538	0.231	0.389	0.143	0.000
